# Burden of and factors associated with poor quality antibiotic, antimalarial, antihypertensive and antidiabetic medicines in Malawi

**DOI:** 10.1371/journal.pone.0279637

**Published:** 2022-12-27

**Authors:** Francis Kachidza Chiumia, Happy Magwaza Nyirongo, Elizabeth Kampira, Adamson Sinjani Muula, Felix Khuluza

**Affiliations:** 1 Department of Pharmacy, Kamuzu University of Health Sciences, Blantyre, Malawi; 2 Department of Medical Laboratory Sciences, Kamuzu University of Health Sciences, Blantyre, Malawi; 3 Department of Public Health, Kamuzu University of Health Sciences, Blantyre, Malawi; University of Turin, ITALY

## Abstract

**Objective:**

To assess the prevalence and factors associated with substandard and falsified (SF) medicines among antibiotic, antimalarial, antihypertensive and antidiabetic medicines in Malawi.

**Methods:**

We conducted a cross-sectional study in 23 public, faith-based and private health facilities in Zomba, Machinga and Nsanje districts. We analyzed oral medicine samples of commonly used medicines among antibiotics, antimalarial, antihypertensive and antidiabetics in accordance with Malawi Essential Medicines List and local treatment guidelines. These medicines were subjected to visual inspection for any defects and screening for the content of active pharmaceutical ingredient and disintegration of dosage units. Samples that failed during screening and at least 10% of those that passed were subjected to pharmacopeia assay and dissolution test for confirmation. We used thin layer chromatography and disintegration test methods provided in the Global Pharma Health Fund minilab^®^ for the screening purposes. We conducted confirmatory test using High-Performance Liquid Chromatography (HPLC) or ultra-violet/visible spectrophotometer and dissolution.

**Results:**

Of the 293 medicine samples collected, 14.3% were SF medicines. Among the SF medicines were 12.5% of Amlodipine (1/8), 19.2% of Amoxicillin (5/26), 72.2% of Atenolol (8/11), 21.2% of Ciprofloxacin (7/33), 14.3% of Enalapril (1/7), 44.4% of Flucloxacillin (4/9), and 35.7% of sulfadoxine/ pyrimethamine (10/28). Medicine quality was associated with therapeutic medicine class, stated origin of manufacturer, primary packaging material and geographical location. Antimalarial and antidiabetic medicines were of better quality as compared to antibiotics, odds ratio OR 4.2 (95% CI 1.7–9.49), p < 0.002 and OR 5.6 (95% CI 1.21–26.09), p < 0.028 respectively. In terms of stated country of origin, the prevalence of SF medicines was 30% (15/50), 33% (9/27), 26.7% (4/15) and 6.6% (8/122) for medicines stated to be manufactured in Malawi, China, Kenya and India respectively.

**Conclusion:**

This study presents the first findings on the assessment of quality of medicines since the establishment of the national pharmacovigilance center in 2019 in Malawi. It is revealed that the problem of SF medicines is not improving and hence the need for further strengthening of quality assurance systems in Malawi.

## Introduction

Substandard and falsified (SF) medicines are a global public health concerns [1]. Substandard medical products are also referred to as ‘out of specification’ products [2]. These are “authorized medical products that fail to meet either their quality standards or specifications or both” [3]. This is caused by limitations during manufacturing processes or post-production degradation [4]. On the other hand, falsified medical products are “medical products that deliberately or fraudulently misrepresent their identity, composition or source” [3]. Undesirable characteristics of SF medicines such as inadequate API content, may lead to harmful effects such as lack of response to treatment, adverse drug events, mortality, and antimicrobial resistance [5].

Some recent studies have started linking adverse health outcomes to the prevalence of SF medicines. A recent systematic analysis by Torloni et.al attributed the high maternal mortality in low and middle-income (LMIC) countries to the high prevalence of SF medicines used for treatment of life-threatening pregnancy complications such as eclampsia, post-partum hemorrhage and sepsis [6]. A 2017 World Health Organization review established that 169,000 children suffering from pneumonia are estimated to die every year as a result of treatment failure due to use of SF antibiotic medicines [7]. About 4% of malarial deaths among under-five patients who receive treatment in sub-Saharan Africa are also reported to occur due to use of SF medicines [8]. Furthermore, SF medicines are also associated with loss of public trust and increased individual, household or health system costs [9].

WHO estimated the prevalence of SF medicines at 10.5% in 2017 for LMIC [2]. The burden is high for African countries with a prevalence of 18.9%. The prevalence for Asian countries is 10.2%,while for other countries, the prevalence is as low as below 1% [10]. Studies done in Malawi between 2014 and 2017 have identified substandard analgesics, uterotonics, antimalarials and antibiotics in public, faith-based, private health facilities and unauthorized vendors. The median prevalence of substandard medicines was 24.7% and the range was 12.5%–88.4% [11–14]. There has also been report of falsified tablets collected from the informal markets that were mislabeled as sulphadoxine/pyrimethamine but contained paracetamol and co-trimoxazole [15].

The prevalence of SF medicines is dynamic [16]. It responds to a number of factors such as disease burden, standard treatment guidelines, and regulatory policies and enforcement [17]. The Malawi Pharmacy and Medicines Regulatory Authority (PMRA) established the national pharmacovigilance center and became a full member of the WHO programme for international drug safety monitoring (WHO-PIDM) in 2019 [18]. The goal of the WHO-PIDM is to reduce medicine-related safety problems including SF medicines through post-marketing quality monitoring and reporting [19]. However, there was limited data to assess for improvements in prevalence of SF medicines in Malawi since 2019. Moreover, the factors associated with the quality of medicines in Malawi had not been investigated.

In this study, we aimed to comparatively assess the current burden of SF medicines among the most commonly used medicines for treatment of both infectious and non-communicable diseases. Hence, we focused on antibiotics, antimalarials, antihypertensives and antidiabetic medicines [20,21]. We also further assessed the factors that are associated with the quality of these medicines.

## Materials and methods

This was a cross-sectional study conducted between July 2021 and January 2022 in 29 health facilities. The health facilities were selected in the southern region of Malawi (Zomba, Machinga and Nsanje districts) using RAND function of Excel. We adapted the guidelines for medicine quality assessment and reporting (MEDQUARG) [22]. Primary healthcare facilities were selected based on stratified random sampling. For each district, the facilities were grouped into public, faith-based and private health facility. We used the RAND function in excel to select four primary health care facility from each group. Where the facilities were ≤ 4, all of them were included in the study. Purposively, we also included each district hospital in the study for comparison. Ordinarily, each district in Malawi has one major hospital that is considered as public facility for secondary level of health care.

Furthermore, all health facilities are clustered into zones, for which each zone has one major public central hospital, which is considered as a tertiary level facility for the zone. The district health office for Zomba district only offers administrative services and bulk storage of the medicines and medical supplies for distribution to the primary health-care facilities, but does not provide patient care services. Hence, we co-opted a central hospital for the zone, which is coincidentally located within Zomba district. By these categories, there were 11 faith-based not for profit, 16 public and two private health facilities at all levels of care. Of these, 23 are primary, five are secondary and one is a tertiary level health-care facility. The primary level health-care facilities are 21 rural health-centres and two private retail pharmacies while the secondary level health-care facilities are the three major public facilities for each of the study district and two community hospitals owned by faith-based organizations. As already highlighted, the only tertiary level facility in the study is a public facility.

In total, 293 medicine samples of commonly used medicines among antibiotics, antimalarials, antihypertensives and antidiabetics were sampled (see S1 Table). The selection of the medicines was in accordance with the Malawi Essential Medicines List (MEML) and the Malawi Standard Treatment guidelines (MSTG) of 2015, which was the one in use at the time of study. The use of MSTG/MEML in the selection of medicines was supplemented with the 150 Central Medical Stores Trust (CMST) Tracer list of items. The 150 CMST tracer list of items is a compilation of 150 items that must be available all the times whenever a customer (health facility) orders medicines. We included oral medicines used as first line or alternative treatments for common bacterial infections, malaria, hypertension and diabetes mellitus type II in Malawi [23]. For antimalarial medicines, further guidance was sourced from the Malawi National Malaria Guidelines 2013 [24] that resulted in addition of Malawi’s second line antimalarial medicine (artesunate/amodiaquine) and first line antimalarial for pregnant women (Quinine oral tablets). As summarized in Table 1 of results section, the selected medicines were Amoxicillin (n = 26), Azithromycin (n = 11), Cefuroxime (n = 1), Ciprofloxacin (n = 33), and Flucloxacillin (n = 9) among antibiotics, Quinine (n = 1), fixed dose combinations of Artemether/ Lumefantrine (n = 99), Artesunate/Amodiaquine (n = 1), and sulfadoxine/pyrimethamine (= 25) among antimalarials, Amlodipine (n = 8), Atenolol (n = 11), Enalapril (n = 7), methyldopa (n = 9) and Hydrochlorothiazide (n = 14) among antihypertensives and Metformin (n = 18) and Glibenclamide (n = 17) among antidiabetic medicines. From the selected medicines, the only child friendly formulation in our sample was dispersible artemether/lumefantrine.

**Table 1:** Characteristics of medicine samples.

Variable	Characteristic	MEML category^a^	Public Health Facilities (N = 16) [189 samples]	CHAM facilities (N = 11) [79 samples]	Licensed retail pharmacies (N = 2) [25 samples]	Total Number of Samples	Samples tested using visual/TLC testing methods	Samples tested according to pharmacopoeia monographs
Medicine type (International Nonproprietary Names)	Amlodipine 5 and 10mg tablet	DVA	3	3	2	8	8	0
	Amoxicillin 250 or 500mg capsule/tablet	HVA	17	8	1	26	26	14
	Artesunate/Amodiaquine 100mg/270mg tablet	DVA	0	1	0	1	1	
	Atenolol 50/100mg tablet	DVA	8	2	1	11	11	11
	Azithromycin 250/500mg tablet	DEA	7	2	2	11	11	0
	Cefuroxime 500mg tablet	N/A	0	0	1	1	1	0
	Ciprofloxacillin 250/500mg tablet	DVA	24	7	2	33	33	15
	Enalapril 5/10mg tablet	DVA	4	1	2	7	7	0
	Flucloxacillin 250mg capsule	DVA	7	0	2	9	9	0
	Glibenclamide 5mg tablet	DVA	12	4	1	17	17	0
	Hydrochlorothiazide 25mg tablet	DVA	7	6	1	14	14	0
	Lumefantrine/Artemether 120mg/20mg tablet	HVA	67	30	2	99	99	0
	Metformin 500mg tablet	DVA	12	2	4	18	18	14
	Methyldopa 250mg tablet	DEA	5	3	1	9	9	0
	Quinine sulfate 300mg tablet	DVA	0	0	1	1	1	0
	Sulfadoxine/pyrimethamine 500mg/25mg tablet	HVA	16	10	2	28	28	10
Total medicine number of samples			189	79	25	293	293	64

^a^ The Malawi Essential Medicines List (MEML) of 2015 specifies the level of health institution at which the medicine is normally permitted for use: H = at health centre, district hospital and central hospital levels; D = at district hospital and central hospital levels only; C = at central hospital level only. N = level of use not specified. The ‘therapeutical priority’ code categorizes medicines based on therapeutic importance of each medicine by the use of: V = vital medicines which are potentially life-saving, of major public health relevance and having significant withdraw side-effects, E = essential medicines which are effective against less severe, but nonetheless significant forms of illness; N = non-essential medicines which are used for minor self-limiting illness and are often of questionable efficacy. The third categorization of ‘procurement system’ has two codes: ‘A’ = medicines required by a large number of patients as such to be routinely procured and stocked by CMST; and ‘B’ = medicines required for a limited number of patients and not routinely stocked by CMST).

Basic information of the medicines such as trade name, strength of dosage units, batch numbers, expiry date, and stated manufacturer were collected from the labeling on the primary or secondary packages using a data collection form. In order to acquire the minimum amount of dosage units for conducting each of the required pharmaceutical tests, we targeted to collect at least 80 dosage units from the sample container and transported the samples to the Kamuzu University of Health Sciences for quality analysis. Where the available stock at facility was low, fewer but more than 20 dosage units were collected. All the dosage units per sample were subjected to visual inspection. Nine dosage units were subjected to screening of which three were analyzed using thin layer chromatography (TLC) and six were subjected to disintegration test. For confirmation, 20 dosage units were subjected to pharmacopeia assay for absolute determination of content of active pharmaceutical ingredient (API) and three for dissolution.

Firstly, we visually inspected the medicines for presence of any visual defects such as non-uniformity of color, size and shape, or presence of breakages and any contaminants. For screening purposes, we conducted semi-quantitative analysis of the content of API using thin layer chromatography (TLC) according to the Global Pharma Health Fund (GPHF) minilab^®^ protocols test [25], and disintegration test as outlined in pharmacopoeia. Medicine samples that failed to comply during screening tests were subjected to pharmacopeia assay and dissolution analysis for confirmation. In addition, we ensured that at least 10% of the remaining samples that complied upon screening were also confirmed by assay and dissolution. These samples were randomly selected according to medicine type, based on the availability of reagents and reference standards. We used Agilent^®^ 1120 high performance liquid chromatography (HPLC) or Biobase^®^ Ultra-violet/ Visible (UV/Vis) spectrophotometry to determine the content of API while PharmaTest^®^1200 dissolution apparatus was used to perform dissolution analysis according to methods provided in the British or Indian Pharmacopoeia [26,27].

A medicine sample was considered substandard if the sample dosage units failed to meet the specifications for either content or release of the API. For TLC, triplicate test samples were spotted and compared with standard spotting of both 80% and 100% concentration on the same plate. A sample failed the test if at least one of the sample spotting presented a difference in terms of travel distance, size and intensity of spot with reference to the standard spotting. Six dosage units were run for disintegration test. All the dosage units were required to disintegrate within 30 minutes to pass the test. For the assay and dissolution tests, we used the specifications provided in the US pharmacopeia for determining whether the medicine was substandard or complied to the specifications [4,28]. Overall, we considered a medicine as substandard if the sample was non-compliant to either the specifications for the API content or dissolution upon confirmation. For a few samples including Flucloxacillin (n = 3), Amlodipine (n = 1) and Enalapril (n = 1), the pharmacopeia confirmation was not performed due to unavailability of reference standards.

Descriptive statistics were used for determining the prevalence of SF medicines. Chi square test was used for comparison of the test results among different variables. The extent to which variables were associated with the quality of the medicines was analyzed using logistic regression.

### Ethical considerations

This study was given ethical clearance by the College of Medicine Research and Ethics Committee (COMREC) under approval number P.11/20/3199. We also sought approval from the Pharmacy and Medicines Regulatory Authority (PMRA) and the directorates of health and social services (DHSS) in all the three participating districts before data collection. Furthermore, during medicine sample collection, consent was sought from each respondent. In the majority of cases the respondent was also the in-charge of the facility. The respondents were provided with written participant information sheet and an oral explanation about the study before signing the consent forms. The participants in this study were mostly health workers employed by various institutions and were above eighteen years of age. No personal data was recorded during this study.

## Results

### Characteristics of medicine samples

**Fig 1:**
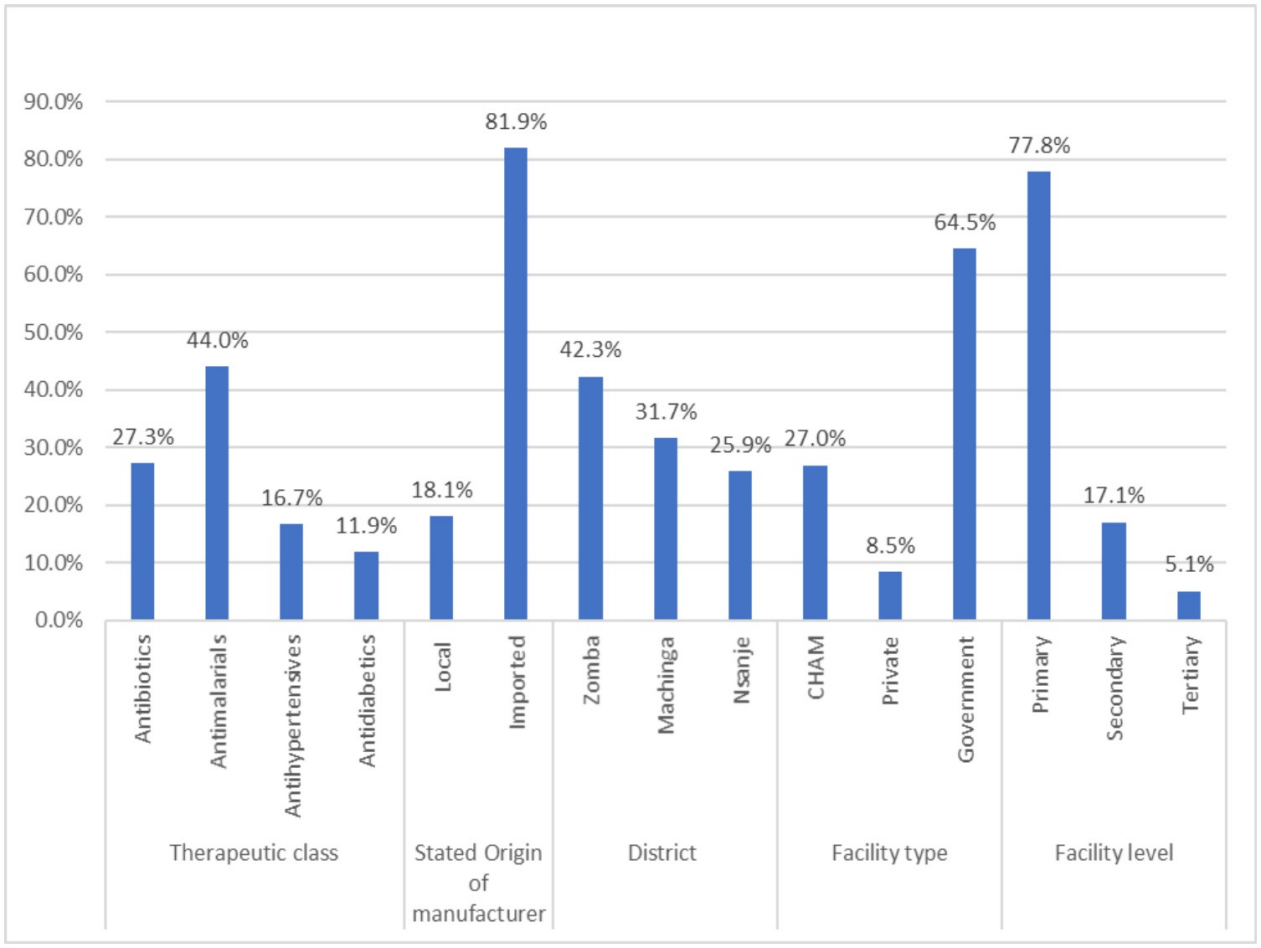
Showing samples tested based on category.

**Fig 2:**
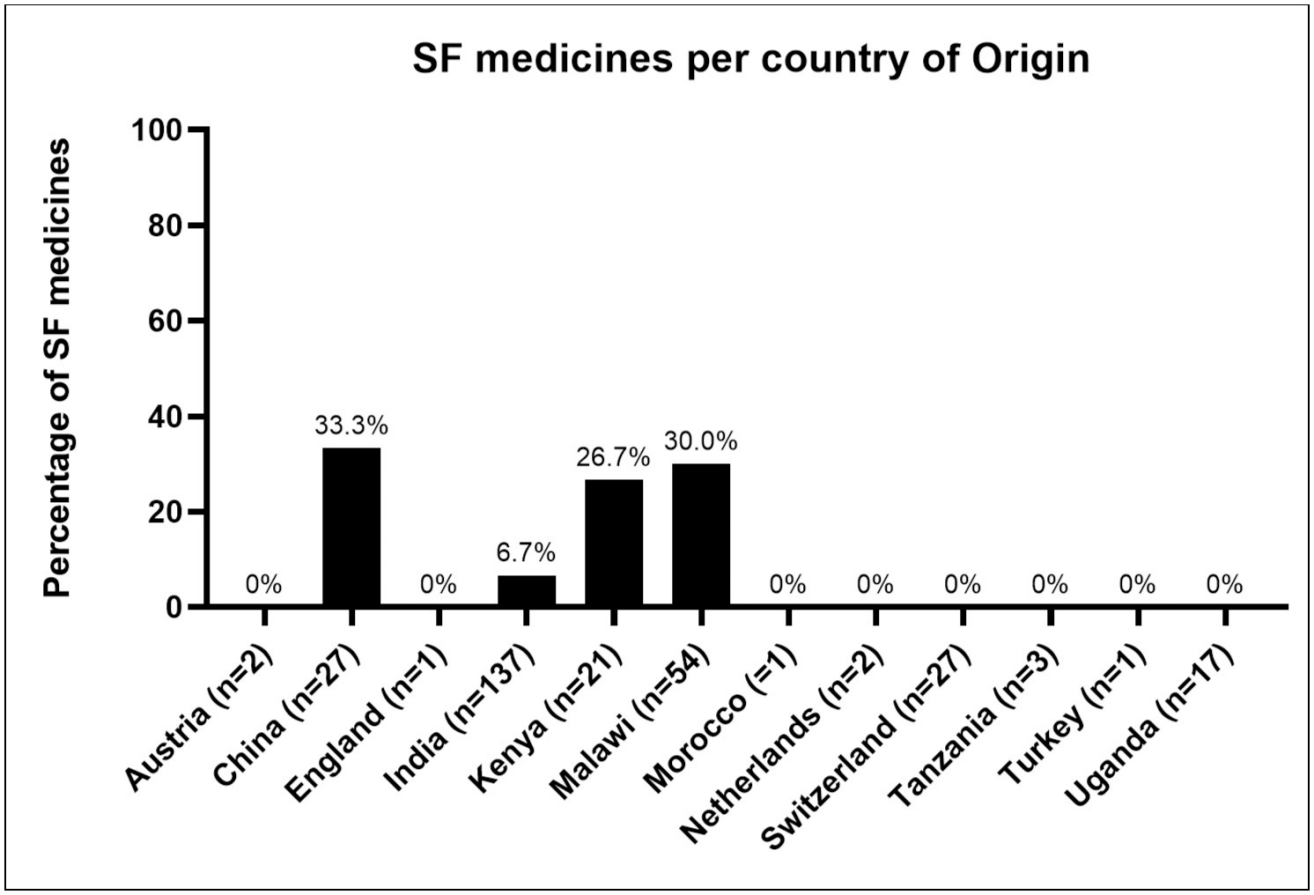
Showing stated country of origin of SF medicines.

Of the 293 medicine samples collected (Table 1, Fig 1), 27.3% (n = 80) were antibiotics: Amoxicillin (n = 26), Azithromycin (n = 11), Cefuroxime (n = 1), Ciprofloxacin (n = 33), and Flucloxacillin (n = 9), 44% (n = 129) were antimalarials: Quinine (n = 1), fixed dose combinations of Artemether/ Lumefantrine (n = 99), Artesunate/Amodiaquine (n = 1), and sulfadoxine/pyrimethamine (= 25), 16.7% (n = 49) were antihypertensives: Amlodipine (n = 8), Atenolol (n = 11), Enalapril (n = 7), methyldopa (n = 9) and Hydrochlorothiazide (n = 14) and 12% (n = 35) were antidiabetic medicines, which were Metformin (n = 18) and Glibenclamide (n = 17). About 17.5% (n = 53) of the samples were claimed to be locally manufactured while 82.5% (n = 240) were claimed to be imported from various countries as indicated in Fig 2; Austria (n = 2), China (n = 27), England (n = 1), India (n = 137), Kenya (n = 21), Morocco (n = 1), Netherlands (n = 2), Switzerland (n = 27), Tanzania (n = 3), Turkey (n = 1), and Uganda (n = 17). In terms of health facilities, 27% (n = 79) of the samples were from faith-based health facilities, 8.5% (n = 25) from private pharmacies and 64.5% (n = 189) from public health facilities. The majority of the samples (77.8%, n = 228) were from health centres which provide primary health care services and mostly found in rural settings (Fig 1).

### Prevalence of SF medicines

All the 293 medicine samples collected were subjected to visual inspection and disintegration. Based on availability of reference standards, 262 samples were further screened using thin layer chromatography (TLC), which performs semi-quantitative analysis of the API content. Of the medicines samples that were fully screened, 14.3% (n = 38) were found to be substandard medicines (Table 2). The substandard medicines were 12.5% of Amlodipine (1/8), 19.2% of Amoxicillin (5/26), 72.2% of Atenolol (8/11), 21.2% of Ciprofloxacin (7/33), 14.3% of Enalapril (1/7), 44.4% of Flucloxacillin (4/9), and 35.7% of sulfadoxine/ pyrimethamine (10/28). The SF medicines were associated with medicine type (by generic name) (p < 0.001), therapeutic class of the medicines (p < 0.001), stated country of origin (p < 0.001) and district where these medicine samples were collected (p <0.042). The statistics were adjusted based on the new denominator.

**Table 2:** Prevalence of out-of-specification medicines.

Variable	Characteristic	Number of samples	Visual analysis, n (%)	TLC, n (%)	Disintegration, n (%)	Dissolution, n (%)	Pharmacopoeia Assay, n (%)	Overall non-compliance rate, n (%)
Medicine type (INN)	Amlodipine 5mg or 10mg tablet	8	0(0)	1 (12.5)	0 (0)			1 (12.5)
	Amoxicillin 250mg/500mg tablet/capsule	26	0 (0)	0 (0)	5 (19.2)	5 (35.7)	5 (35.7)	5 (19.2)
	Artesunate/Amodiaquine	1	0 (0)	-	0 (0)			
	Atenolol	11	6 (54.6)	0 (0)	0 (0)	5 (45.5)	4 (36.4)	8(72.7)
	Azithromycin	11	0 (0)	-	0 (0)			-
	Cefuroxime	1	0 (0)	-	0 (0)			-
	Ciprofloxacillin	33	0 (0)	3 (9.1)	4 (12.1)	2 (20)	6 (42.9)	7(21.2)
	Enalapril	7	0 (0)	1 (14.3)	0 (0)			1 (14.3)
	Flucloxacillin	9	0 (0)	-	4 (44.4)			4 (44.4)
	Glibenclamide	17	0 (0)	0 (0)	0 (0)			0 (0)
	Hydrochlorothiazide	14	0 (0)	0 (0)	0 (0)	0 (0)		0 (0)
	Lumefantrine/Artemether	99	0 (0)	0 (0)	0 (0)			0 (0)
	Metformin	18	1 (5.6)	0 (0)	0 (0)	0 (0)	2 (11.1)	2 (11.1)
	Methyldopa	9	0 (0)	0 (0)	0 (0)			-
	Quinine sulfate	1	0 (0)	0 (0)	0 (0)			-
	Sulfadoxine/pyrimethamine	28	0 (0)	2 (7.1)	3 (10.7)		10 (100)	10 (35.7)
Therapeutic class	Antibiotics	80	0 (0)	3 (5.1)	13 (16.3)	7 (29.2)	11 (39.3)	16 (25.4)
	Antimalarials	129	0 (0)	2 (1.2)	3 (2.3)		10 (100)	10 (7.8)
	Antihypertensives	49	6 (12.2)	2 (5)	0 (0)	5 (26.3)	4 (36.4)	10 (25)
	Antidiabetics	35	1 (2.9)	0 (0)	0 (0)	0 (0)	2 (11.1)	2 (5.7)
Stated Origin of manufacturer	Local	53	7 (13.2)	3 (6.1)	6 (11.3)	9 (37.5)	10 (34.5)	15 (30.6)
	Imported	240	0 (0)	4 (1.9)	10 (4.2)	3 (9.7)	17 (44.7)	23 (10.6)
District	Zomba	124	5 (4.1)	2 (1.9)	8 (6.5)	7 (29.1)	13 (37.1)	19 (17.3)
	Machinga	93	0 (0)	1 (1.2)	3 (3.2)	2 (13.3)	6 (40)	7 (8.3)
	Nsanje	76	2 (2.7)	4 (5.6)	5 (6.6)	3 (18.8)	8 (47.1)	12 (16.7)
Facility type	Government	189	6 (3.2)	6 (3.5)	10 (5.3)	9 (23.7)	20 (41.7)	27 (15.7)
	CHAM	79	1 (1.3)	1 (1.4)	4 (5.1)	3 (20)	6 (46.2)	8 (11)
	Private	25	0 (0)	0 (0)	2 (8)	0 (0)	1 (16.7)	3 (14.3)
Facility level	Primary	228	3 (1.3)	7 (3.4)	13 (5.7)	6 (17.1)	20 (44.4)	11 (5.7)
	Secondary	50	3 (6)	0 (0)	3 (6)	5 (29.4)	6 (31.6)	27 (13)
	Tertiary	15	1 (6.7)	0 (0)	0 (0)	1 (33.3)	1 (33.3)	2 (16.7)
All	NA	293	7 (2.4)	7 (2.7)	16 (5.5)	12 (21.8)	27 (40.3	38 (14.3)
**Total number of samples**		**293**	**293**	**262**	**293**	**55**	**67**	

Of the 38 SF medicines, 32 samples were confirmed using through pharmacopeia assay or dissolution test (see S2 Table). Except for sulfadoxine/pyrimethamine (SP) tablets, all the samples presented with non-extreme deviations from the acceptable ranges. For the ten SP samples, there was notably higher than the declared content (more than 120% of label claim) of pyrimethamine while sulfadoxine was available in extremely lower amounts (below 80% of label claim) for all the samples subjected to HPLC assay. Apparently, all the SP samples were stated to be manufactured by the same one company from China. Of special note is that the purportedly names of the manufacturers as extracted from the packaging have been omitted to ensure anonymity. However, for all the samples that failed confirmatory tests, we reported to the Pharmacy and Medicines Regulatory Authority with the details of the manufacturers and where the medicines were sampled for further action.

### Factors associated with substandard (quality of) medicines

Results of the logistic regression analysis for possible factors affecting quality of medicines such as therapeutic class, stated origin of manufacturer, primary packaging material, storage stores, and location of health facility are presented in Table 3. Odds ratios (OR) for complying to quality specifications were compared with antibiotic medicine class, local manufacturing, bottle primary packaging, ordinary storage stores and Zomba district respectively as reference characteristics.

**Table 3:** Factors associated with SF medicines in southern Malawi.

			Compliance to Quality specifications		
Variable	Comparator	Characteristic	OR	95% Conf. Interval	P value
Therapeutic class	Antibiotics	Antimalarials	4.2	1.7–9.49	0.002
		Antihypertensives	1.02	0.41–2.54	0.964
		Antidiabetics	5.6	1.21–26.09	0.028
Stated country of Origin for manufacturer	Local	Imported	3.72	1.78–7.84	0.001
Primary packaging	Bottle	blisters	5.05	2.33–10.91	<0.000
Storage	Ordinary drug store	SIAB	0.84	0.42–1.69	0.63
District	Zomba	Machinga	2.29	1.02–6.74	0.026
		Nsanje	1.04	0.47–2.31	0.915

Medicine samples from Machinga districts had better-quality medicines, OR 2.29 (95% CI 1.02–6.74), p< 0.026) as compared to those sampled from Zomba district while there was no significant difference with those sampled from Nsanje district, OR 1.04 (95% CI 0.47–2.31), p < 0.915.

In terms of therapeutic medicine class, antibiotic medicines had the highest rate of non-compliance to quality specifications after adjustment basing on confirmatory tests. Antidiabetic medicines had the highest quality OR 5.6 (95% CI 1.21–26.09), p < 0.028 and were seconded by antimalarial medicines with OR 4.2 (95% CI 1.7–9.49), p < 0.002. The quality of antihypertensive medicines was comparable to antibiotic medicines with OR 1.02 (95% CI 0.41–2.54), p < 0.964. We further analyzed the quality of the medicines by therapeutic class per district and found that only results for antimalarial medicines were significant in Nsanje district, p < 0.003. The rest of the medicine classes per each district, except antihypertensives in Zomba, had OR > 1 in comparison with antibiotics but the results were not statistically significant.

We also found imported medicines to have better quality in comparison with locally manufactured medicines, OR 3.72 (CI 1.78–7.84), p < 0.001 and also medicines primarily packed in blister packs were of better quality as compared to medicines packaged directly in bottles, OR 5.05 (95% CI 2.33–10.91), p < 0.000. On further stratified analysis per district, the good quality of the medicines for imported medicines was significant for Zomba and Nsanje districts, OR 2.75 (95% CI 1.3–7.8), p < 0.046 and OR 6.5 (95% CI 1.67–25.18), p < 0.007. In terms of packaging, the results were significant in Zomba and Machinga with OR 5.7 (95% CI 1.68–17.8), p < 0.005 and OR 5.7 (95% CI 1.04–31.8), p < 0.045 respectively.

### Origin of substandard medicines in Malawi

All the detected substandard medicines were manufactured in Africa and Asian countries **(**Fig 2**)**. There was a significant variation in the proportion of SF medicines among the countries where the medicine samples were manufactured (P< 0.001). Prevalence of SF medicines was rampant for medicines claimed to be locally manufactured (30%), from China (33%), Kenya (26.7%) and India (6.6%).

## Discussion

Routine post-marketing surveillance of medicine quality is not common in resource limited countries such as Malawi [29]. Thus, with respect to the WHO Global Benchmarking tool for evaluation of national regulating systems of medical products, majority of LMICs are at lower levels (level 1 or 2) of maturity as they lack resources to maintain strong and active systems and structures that can enable them to achieve properly documented integrated monitoring activities at various levels. Another major challenge of national medicines regulatory authorities in LMICs is lack of human resource [30]. As such, the quality of medicines on the market remains a concern. Our study found that 14.3% of the antibiotics, antimalarial, antihypertensive and antidiabetic medicines in our sample were substandard based on tests for content and in-vitro release of API. The medicine classes analyzed in the study are crucial as they are used for treatment of diseases with a high morbidity and mortality in Malawi and other LMICs. For instance, more than 50% of prescriptions in LMICs contain antibiotic medicines [31]. On the other hand, Malaria is one of the leading cause of death in Malawian children under the age of five years [32]. It is also revealed that the burden of hypertension and diabetes is increasing in Malawi [33]. Therefore, the compromised quality of medicines analyzed in this study have potential to cause hazardous effects on the public health.

Previous studies done in Malawi found SF medicine prevalence of 45.5% and 13% in 2014 and 2017 respectively among antibiotic medicines. In these studies, the sampled antibiotics included co-trimoxazole, phenoxymethylpenicillin, Ciprofloxacin, Amoxicillin/Clavulanic acid, chloramphenicol and Cefuroxime [11,13]. In this study, we sampled Amoxicillin, Azithromycin, Cefuroxime, Ciprofloxacin and Flucloxacillin and found a prevalence of SF medicines of 25.4%. This is 1.79-fold lower than the prevalence in 2014 but 1.9-fold higher than results if the study done in 2017. The continued high prevalence of SF antibiotics is worrisome for low-income countries such as Malawi. This is based on the fact that the country is burdened with high infectious diseases which requires quality treatment using antibiotics. The poor-quality antibiotics may therefore contribute to mortality and antimicrobial resistance due to under-dosing in individual patients.

In 2015, a study by Chikowe et.al found a very high prevalence of 88.8% substandard antimalarial medicines [12] while in 2017 study by Khuluza et.al the prevalence of SF medicines was 9% among antimalarial medicines [13]. Both studies included samples artemether/lumefantrine, Sulphadoxine/ Pyrimethamine, Quinine and Artesunate/ Amodiaquine medicines and these were also included our study. In the current findings, we found a slightly lower prevalence (7.8%) than the 2017 prevalence of SF medicines among antimalarial medicines. The methods applied in these three studies are similar and therefore the variations might be likely attributable to times that these studies were conducted and thereby further confirming that the prevalence of SF medicines is dynamic and therefore continuous efforts to curb the burden are necessary.

In the past studies, little attention was given to the quality of medicines for treatment of non-communicable diseases in Malawi and sub-Saharan Africa. In this study, we also included antihypertensive medicines such as Amlodipine, Atenolol, Enalapril, Methyldopa and Hydrochlorothiazide and oral antidiabetic medicines such as Metformin and Glibenclamide. The prevalence of SF medicines among antihypertensives was 25%, which is comparable to the high prevalence of SF medicines among antibiotics. Antidiabetics on the other hand, have a lower prevalence of 5.7%. Though most studies have found that antimalarial and antibiotics are mostly to be substandard and falsified in low-income countries, this study has found that the problem is also prevalent in non-communicable disease (NCD) medicines, especially the antihypertensives. This requires strong post marketing surveillance system targeting medicines for treatment of all medicines regardless of the disease that they are intended to treat.

The factors associated with SF medicines in this study were therapeutic medicine class, stated country of origin, primary packaging material, and geographical area. Previous studies have shown that there is disproportionality in the burden of SF medicines across the globe, with low-income countries recording a high prevalence of SF medicines used for treatment of infectious diseases such as antibiotics while in high-income countries, the burden is low and mostly concern expensive medicines for lifestyle use such as steroids and treatments erectile dysfunction [10,34]. Further, it is postulated that economic factors such as high market demand as propelled by the disease burden, play a role in proliferation of SF medicines [17]. Antibiotics and antimalarial medicines are among the most commonly used medicines in Malawi and therefore we expected to have higher prevalence of SF medicines for these therapeutic classes. Our results however, found a high prevalence of SF medicines among antibiotics (25.4%) and a low prevalence of SF medicines among antimalarials (7.8%). The low prevalence of SF medicines among antimalarial medicines is probably due to a more controlled supply chain system for medicines and medical supplies for special public health programs such as Malaria, HIV and Tuberculosis. In Malawian public and faith-based health care facilities, antimalarial medicines are sourced from a limited number of World Health Organization pre-qualified suppliers and the distribution and storage of these medicines is closely monitored. This may be the reason for the observed low prevalence of SF medicines for the antimalarials in the study.

On the other hand, incidence of non-communicable diseases is also escalating in LMICs [20,21]. Consistent with the increased demand, it was observed in the study that there was also high prevalence of SF medicines for antihypertensive medicines (25%). Although the prevalence of SF medicines for antidiabetic medicines was relatively low (5.7%), the current results raise a signal for an emerging concern of the quality of medicines for treatment of non-communicable diseases (NCDs) in Malawi.

Our results revealed that locally manufactured medicines are likely to be of poor quality as compared to imported medicines. This may be attributable to inadequate regulatory supervision on the part of Medicine regulator and lack of adequate resources to enable manufacturing and quality control processes that are fully compliant to current good manufacturing practices (cGMP) in local pharmaceutical companies. An assessment of pharmaceutical quality assurance systems showed that there is poor compliance to the WHO set guidelines for quality assurance in LMICs [35]. This is even likely to be more prominent in countries with relatively weaker regulatory framework such as Malawi [36]. In Malawi, the regulatory authority has put in place strong efforts and guidelines to ensure good quality of medicines. This include active surveillance, raising awareness to health care professionals and ensuring that reporting tools are available for reporting suspected SF medicines in health facilities. However, challenges such as inadequate human and financial resources are affecting such activities.

Blister packaging offers additional protection to tablet and capsule dosage units through prevention of direct contact with atmospheric air, humidity and sunlight; which are the major factors that facilitate the deterioration of medicines on storage [37–39]. Our results provide further evidence on this, as it was found that medicine samples that used blisters for primary packaging were of significantly better quality as compared to those that used bottles for primary packaging. With an annual mean temperature of 28°C (www.worlddata.info/africa/malawi), the southern region of Malawi may be considered as an area with unfavorable conditions for storage of medicines without temperature control mechanisms. The normal acceptable range for storage of most of oral solid dosage forms is 15°C to 25°C, but depends on the manufacturer’s recommendation which must be based on formal stability studies [40]. It is therefore important to emphasize on the need for compliance to storage requirements of the medicines as stated by the manufacturer’s labelling for all medicine types.

Based on the assertion that higher temperatures are more likely to cause a higher degradation rate for medicines, we expected a higher prevalence of poor-quality medicines in Nsanje district, which records the highest temperatures across the year in the southern Malawi. On the contrary, Zomba which relatively records lower temperatures had the highest prevalence of poor-quality medicines. This difference could be likely due to the storage practices of medicines in the health facilities. For instance, despite the higher temperatures in the district, 77% of the study sites in Nsanje district use the ‘Storage in a box’ (SIaB) units while only 43% and 32% of study facilities in have the SIaB in Zomba and Machinga respectively. SIAB units are prefabricated medicine storage rooms designed to easily control temperature by installing them using insulated wall panels, double roofing and heat reflector painting. The SIaB units are a USAID initiative, which was aimed at improving the storage conditions and capacity for health facilities in resource-limited places such as Malawi. These storage units offer better conditions than ordinary medicine stores as they are pre-also contain air conditioners and built-in room thermometers for continuous monitoring of the daily storage conditions [41].

The factors associated with SF medicines provides insights into possible challenges of the supply chain system in Malawi. The Central Medical Stores Trust (CMST) procures, stores and distributes medicines and medical supplies for public and faith-based health institutions in Malawi [42]. This centralized system is advantageous in resource limited settings like Malawi as it provides a single route of entry of medicines into the supply chain which can be easily monitored for any possible quality defects. It is therefore important to strengthen the measures for countering SF medicines from the CMST level down the supply chain system. For medicines that are supplied outside the CMST system, the same level of regulatory supervision need to be established and implemented across the country, to avoid that there are different standards between the public and private sector.

### Limitations of the study

In this study, not all the medicine samples were subjected to pharmacopeia analysis; HPLC and UV-VIS spectrometry for analysis of API content and dissolution tester for analysis of the release of API which are considered as gold standard techniques. However, we ensured that at least 10% of samples and those that failed upon TLC and disintegration testing were confirmed by the pharmacopeia methods. As highlighted, a few medicines that failed the screening stage, were not verified by the pharmacopeia analysis. This is due to lack of reference standards due to procurement challenges as the study was done when COVID-19 pandemic was at the peak in Malawi.

## Conclusion

This study presents the first results on the assessment of quality of medicines since the establishment of the national pharmacovigilance centre in 2019 in Malawi. It is revealed that the problem of SF medicines is not improving and therefore the measures that are implemented in order to curb this problem needs further strengthening. It is also recommended that the capacity of the National Regulatory Authority is strengthened, for better carrying out essential regulatory tasks including post marketing surveillance.

## Supporting information

S1 Table List of all medicines collected for the study. (DOCX)

S2 Table Description of all medicines that failed test. (DOCX)
