# Supplementary material for: Burden of and factors associated with poor quality antibiotic, antimalarial, antihypertensive and antidiabetic medicines in Malawi
Source: PLoS One. 2022 Dec 27;17(12):e0279637. doi: 10.1371/journal.pone.0279637 (PMC9794066; doi:10.1371/journal.pone.0279637)
Supplement: S1 Table — (DOCX) [file pone.0279637.s001.docx]

**S1 Table: List of all medicines collected for the study on Burden of and factors associated with poor quality antibiotic, antimalarial, antihypertensive and antidiabetic medicines in Malawi**

| **Sample ID** | **Generic name** | **Strength** | **MEML category^a^** | **Stated Country of origin** | **Expiry date** | **Storage** | **Primary Pack** | **Visual** | **Disintegration** | **Dissolution** | **TLC** | **Assay** |
| --- | --- | --- | --- | --- | --- | --- | --- | --- | --- | --- | --- | --- |
| NS TRI 285 | Amoxicillin | 250mg | HVA | Kenya | Mar-24 | SIAB | Bottle | Pass | Pass |  | Pass |  |
| MG DHO 142 | Amoxicillin | 250mg | HVA | Malawi | Feb-24 | Ordinary | Bottle | Pass | Pass |  | Pass |  |
| MG NAY 52 | Amoxicillin | 250mg | HVA | Malawi | Dec-23 | SIAB | Bottle | Pass | Fail | Fail | Fail | Fail |
| MG MPI 68 | Amoxicillin | 250mg | HVA | India | Apr-22 | Ordinary | Bottle | Pass | Fail | Fail | Fail | Fail |
| MG NAM 59 | Amoxicillin | 250mg | HVA | Malawi | Oct-23 | SIAB | Bottle | Pass | Pass | Pass | Pass | Pass |
| ZA MAC 181 | Amoxicillin | 250mg | HVA | Malawi | Jan-24 | SIAB | Bottle | Pass | Pass | Pass | Pass | Pass |
| MG MPO 108 | Amoxicillin | 250mg | HVA | Kenya | Jun-22 | Ordinary | Bottle | Pass | Pass | Pass | Pass | Pass |
| NS SAN 255 | Amoxicillin | 250mg | HVA | Malawi | Nov-23 | SIAB | Bottle | Pass | Pass | Pass | Pass | Pass |
| NS TRI 244 | Amoxicillin | 250mg | HVA | Kenya | Jun-22 | SIAB | Bottle | Pass | Fail | Fail | Fail | Fail |
| ZA NKA 01 | Amoxicillin | 250mg | HVA | Kenya | Jun-22 | Ordinary | Bottle | Pass | Pass | Pass | Pass | Pass |
| ZA DHO 22 | Amoxicillin | 250mg | HVA | Malawi | Dec-23 | Ordinary | Bottle | Pass | Pass | Pass | Pass | Pass |
| ZA STL 117 | Amoxicillin | 25mg | HVA | India | Jul-22 | SIAB | Bottle | Pass | Pass | Pass | Pass | Pass |
| ZA STL 118 | Amoxicillin | 250mg | HVA | Kenya | Jun-22 | SIAB | Bottle | Pass | Fail | Fail | Fail | Fail |
| NS KAL 237 | Amoxicillin | 250mg | HVA | India | May-22 | SIAB | Bottle | Pass | Pass |  | Pass |  |
| ZA CTL 032 | Amoxicillin | 250mg | HVA | Austria | Feb-22 | Ordinary | Bottle | Pass | Pass | Pass | Pass | Pass |
| MG MED 094 | Amoxicillin | 625mg | HVA | India | Feb-23 | Ordinary | Bottle | Pass | Pass |  | Pass |  |
| NS DHO 196 | Amoxicillin | 250mg | HVA | Malawi | Oct-23 | Ordinary | Bottle | Pass | Pass | Pass | Pass | Pass |
| ZA CTL 031 | Amoxicillin | 250mg | HVA | Malawi | Aug-23 | Ordinary | Bottle | Pass | Pass |  | Pass |  |
| ZA CTL 033 | Amoxicillin | 625mg | HVA | India | Jul-22 | Ordinary | Bottle | Pass | Pass |  | Pass |  |
| MG NAM 85 | Amoxicillin | 250mg | HVA | Kenya | Jun-22 | Ordinary | Bottle | Pass | Pass |  | Pass |  |
| ZA NKA 02 | Amoxicillin | 250mg | HVA | India | Aug-22 | Ordinary | Bottle | Pass | Pass |  | Pass |  |
| ZA THO 09 | Amoxicillin | 250mg | HVA | Malawi | Oct-23 | SIAB | Bottle | Pass | Fail |  | Fail |  |
| ZA MAT 171 | Amoxicillin | 250mg | HVA | India | Aug-22 | Ordinary | Bottle | Pass | Pass |  | Pass |  |
| ZA CHA 174 | Amoxicillin | 250mg | HVA | Malawi | Nov-23 | SIAB | Bottle | Pass | Pass |  | Pass |  |
| ZA MAY 152 | Amoxicillin | 625mg | HVA | India | May-22 | Ordinary | Bottle | Pass | Pass |  | Pass |  |
| ZA CHA 293 | Amoxicillin | 250mg | HVA | Malawi | Jan-24 | SIAB | Bottle | Pass | Pass |  | Pass |  |
| ZA MAT 162 | Azithromycin | 500mg | DEA | Kenya | Oct-22 | Ordinary | Strips | Pass | Pass |  |  |  |
| MG NAM 60 | Azithromycin | 500mg | DEA | India | Nov-22 | SIAB | Strips | Pass | Pass |  |  |  |
| NS PHO 220 | Azithromycin | 500mg | DEA | Austria | Oct-22 | SIAB | Strips | Pass | Pass |  |  |  |
| NS PHO 221 | Azithromycin | 500mg | DEA | India | Nov-22 | SIAB | Strips | Pass | Pass |  |  |  |
| MG CHI 076 | Azithromycin | 500mg | DEA | India | Nov-22 | SIAB | Strips | Pass | Pass |  |  |  |
| MG MED 096 | Azithromycin | 500mg | DEA | India | Mar-22 | Ordinary | Strips | Pass | Pass |  |  |  |
| ZA MAY 154 | Azithromycin | 500mg | DEA | India | Feb-22 | Ordinary | Strips | Pass | Pass |  |  |  |
| ZA GHE 45 | Azithromycin | 500mg | DEA | Kenya | Oct-24 | Ordinary | Strips | Pass | Pass |  |  |  |
| MG NAY 283 | Azithromycin | 500mg | DEA | India | Dec-23 | SIAB | Strips | Pass | Pass |  |  |  |
| MG GAW 284 | Azithromycin | 500mg | DEA | India | Dec-23 | Ordinary | Strips | Pass | Pass |  |  |  |
| ZA THO 265 | Azithromycin | 250mg | DEA | India | Apr-24 | SIAB | Strips | Pass | Pass |  |  |  |
| MG MED 097 | Cefuroxime | 500mg | N/A | India | Oct-23 | Ordinary | Strips | Pass | Pass |  |  |  |
| ZA CHA 294 | Ciprofloxacin | 250mg | DVA | Malawi | Jan-24 | SIAB | Bottle | Pass | Fail |  | Fail |  |
| ZA MAT 163 | Ciprofloxacin | 500mg | DVA | India | Nov-22 | Ordinary | Strips | Pass | Pass | Pass | Pass | Pass |
| ZA THO 12 | Ciprofloxacin | 500mg | DVA | India | Jun-24 | SIAB | Strips | Pass | Pass | Pass | Pass | Pass |
| MG DHO 144 | Ciprofloxacin | 500mg | DVA | India | Jun-24 | Ordinary | Strips | Pass | Pass | Pass | Pass | Pass |
| MG GAW 135 | Ciprofloxacin | 500mg | DVA | India | Jun-24 | Ordinary | Strips | Pass | Pass |  | Pass |  |
| ZA CHA 176 | Ciprofloxacin | 250mg | DVA | Malawi | Oct-23 | SIAB | Bottle | Pass | Pass |  | Pass |  |
| MG NAM 86 | Ciprofloxacin | 250mg | DVA | India | Aug-23 | Ordinary | Strips | Pass | Pass |  | Pass |  |
| MG NAY 53 | Ciprofloxacin | 250mg | DVA | Kenya | Aug-23 | SIAB | Strips | Pass | Pass |  | Pass |  |
| MG MPI 69 | Ciprofloxacin | 500mg | DVA | Kenya | Sep-22 | Ordinary | Strips | Pass | Pass | Pass | Pass | Pass |
| MG NAM 61 | Ciprofloxacin | 250mg | DVA | India | Jul-23 | SIAB | Strips | Pass | Pass |  | Pass |  |
| MG MPO 109 | Ciprofloxacin | 500mg | DVA | India | Jul-23 | Ordinary | Strips | Pass | Pass |  | Pass |  |
| MG MGH 128 | Ciprofloxacin | 250mg | DVA | India | Aug-23 | SIAB | Strips | Pass | Pass |  | Pass |  |
| NS SAN 256 | Ciprofloxacin | 250mg | DVA | Malawi | Oct-23 | SIAB | Bottle | Pass | Pass |  | Pass |  |
| NS CHI 213 | Ciprofloxacin | 500mg | DVA | India | Jun-24 | Ordinary | Strips | Pass | Pass |  | Pass |  |
| NS TRI 246 | Ciprofloxacin | 500mg | DVA | India | May-23 | SIAB | Strips | Pass | Pass |  | Pass |  |
| ZA NKA 04 | Ciprofloxacin | 500mg | DVA | Netherlands | Feb-22 | Ordinary | Strips | Pass | Pass |  | Pass |  |
| NS MBE 205 | Ciprofloxacin | 500mg | DVA | India | Jun-24 | SIAB | Strips | Pass | Pass | Pass | Pass | Pass |
| ZA DHO 24 | Ciprofloxacin | 250mg | DVA | Malawi | Oct-23 | Ordinary | Bottle | Pass | Pass | Pass | Pass | Pass |
| ZA STL 120 | Ciprofloxacin | 500mg | DVA | India | Jul-23 | SIAB | Strips | Pass | Pass | Pass | Pass | Pass |
| ZA CTL 035 | Ciprofloxacin | 250mg | DVA | Malawi | Oct-23 | Ordinary | Bottle | Pass | Pass |  | Pass |  |
| MG CHI 077 | Ciprofloxacin | 250mg | DVA | India | Aug-23 | SIAB | Strips | Pass | Pass |  | Pass |  |
| MG MED 098 | Ciprofloxacin | 500mg | DVA | India | Jun-23 | Ordinary | Strips | Pass | Pass |  | Pass |  |
| ZA THO 11 | Ciprofloxacin | 250mg | DVA | Malawi | Oct-23 | SIAB | Bottle | Pass | Pass |  | Pass |  |
| ZA GHE 46 | Ciprofloxacin | 500mg | DVA | India | Feb-23 | Ordinary | Strips | Pass | Pass |  | Pass |  |
| MG NAY 282 | Ciprofloxacin | 500mg | DVA | India | Jun-24 | SIAB | Strips | Pass | Pass |  | Pass |  |
| NS MBE 288 | Ciprofloxacin | 250mg | DVA | Malawi | Sep-24 | SIAB | Bottle | Pass | Fail | Fail | Fail | Fail |
| NS MBE 289 | Ciprofloxacin | 500mg | DVA | India | Jun-24 | SIAB | Strips | Pass | Pass | Pass | Fail | Fail |
| NS PHO 290 | Ciprofloxacin | 250mg | DVA | Malawi | Sep-24 | SIAB | Bottle | Pass | Fail | Fail | Fail | Fail |
| NS TRI 286 | Ciprofloxacin | 500mg | DVA | India | Jun-24 | SIAB | Strips | Pass | Pass | Pass | Fail | Fail |
| NS MBE 301 | Ciprofloxacin | 250mg | DVA | Malawi | Sep-24 | SIAB | Bottle | Pass | Pass | Pass | Fail | Fail |
| ZA THO 266 | Ciprofloxacin | 250mg | DVA | India | Sep-24 | SIAB | Strips | Pass | Pass |  | Pass |  |
| ZA MAT 164 | Flucloxacillin | 250mg | DVA | Kenya | Dec-22 | Ordinary | Strips | Pass | Fail |  | Fail |  |
| ZA GHE 47 | Flucloxacillin | 250mg | DVA | Kenya | Jul-23 | Ordinary | Strips | Pass | Fail |  | Fail |  |
| ZA MAC 182 | Flucloxacillin | 250mg | DVA | India | Aug-22 | SIAB | Strips | Pass | Fail |  | Fail |  |
| ZA DHO 25 | Flucloxacillin | 250mg | DVA | India | Aug-22 | Ordinary | Strips | Pass | Pass |  | Pass |  |
| ZA CTL 036 | Flucloxacillin | 250mg | DVA | India | Aug-22 | Ordinary | Strips | Pass | Pass |  | Pass |  |
| MG MED 100 | Flucloxacillin | 250mg | DVA | England | Sep-22 | Ordinary | Strips | Pass | Fail |  | Fail |  |
| ZA MAK 277 | Flucloxacillin | 250mg | DVA | India | Aug-22 | SIAB | Strips | Pass | Pass |  | Pass |  |
| ZA CTL 268 | Flucloxacillin | 250mg | DVA | India | Mar-23 | Ordinary | Strips | Pass | Pass |  | Pass |  |
| ZA DHO 267 | Flucloxacillin | 250mg | DVA | India | Aug-22 | Ordinary | Strips | Pass | Pass |  | Pass |  |
| ZA MAK 189 | Ciprofloxacin | 250mg | DVA | Malawi |  | SIAB | Bottle | Pass | Pass |  | Pass |  |
| NS DHO 257 | Ciprofloxacin | 250mg | DVA | Malawi |  | Ordinary | Bottle | Pass | Fail | Fail | Pass | Pass |
| ZA MAT 160 | Amlodipine | 10mg | DVA | India | Aug-22 | Ordinary | Strips | Pass | Pass |  | Pass |  |
| ZA GHE 44 | Amlodipine | 5mg | DVA | India | Jun-23 | Ordinary | Strips | Pass | Pass |  | Pass |  |
| MG MPO 107 | Amlodipine | 5mg | DVA | India | Apr-22 | Ordinary | Strips | Pass | Pass |  | Pass |  |
| ZA STL 116 | Amlodipine | 5mg | DVA | India | Apr-22 | SIAB | Strips | Pass | Pass |  | Pass |  |
| NS PHO 219 | Amlodipine | 5mg | DVA | India | Sep-21 | SIAB | Strips | Pass | Pass |  | Pass |  |
| NS KAL 236 | Amlodipine | 10mg | DVA | India | Aug-23 | SIAB | Strips | Pass | Pass |  | Fail |  |
| MG MED 093 | Amlodipine | 10mg | DVA | India | Jun-23 | Ordinary | Strips | Pass | Pass |  | Pass |  |
| ZA MAK 188 | Amlodipine | 5mg | DVA | India |  | SIAB | Strips | Pass | Pass |  | Pass |  |
| ZA MAT 161 | Atenolol | 50mg | DVA | Malawi | Sep-22 | Ordinary | Bottle | Pass | Pass | Pass | Fail | Fail |
| MG DHO 143 | Atenolol | 50mg | DVA | India | Sep-22 | Ordinary | Strips | Pass | Pass | Pass | Fail | Fail |
| ZA CHA 175 | Atenolol | 50mg | DVA | Malawi | Mar-23 | SIAB | Bottle | Fail | Pass | Pass | Fail | Fail |
| ZA DHO23 | Atenolol | 50mg | DVA | Malawi | Feb-23 | Ordinary | Bottle | Fail | Fail | Fail | Fail | Fail |
| ZA STL 119 | Atenolol | 50mg | DVA | Malawi | Oct-21 | SIAB | Bottle | Fail | Fail | Fail | Pass | Pass |
| ZA CTL 034 | Atenolol | 50mg | DVA | Malawi | Oct-22 | Ordinary | Bottle | Fail | Fail | Fail | Pass | Pass |
| MG MED 095 | Atenolol | 100mg | DVA | India | Aug-22 | Ordinary | Strips | Pass | Pass | Pass | Pass | Pass |
| NS DHO 201 | Atenolol | 50mg | DVA | Malawi | Oct-22 | Ordinary | Bottle | Fail | Fail | Fail | Pass | Pass |
| ZA NKA 03 | Atenolol | 50mg | DVA | Malawi | Oct-21 | Ordinary | Bottle | Pass | Pass | Pass | Pass | Pass |
| ZA MAY 153 | Atenolol | 50mg | DVA | India | Sep-22 | Ordinary | Strips | Pass | Pass | Pass | Pass | Pass |
| ZA THO 10 | Atenolol | 50mg | DVA | Malawi | Mar-23 | SIAB | Bottle | Fail | Fail | Fail | Pass | Pass |
| ZA MAT 170 | Enalapril | 10mg | DVA | India | Jun-23 | Ordinary | Strips | Pass | Pass |  | Pass |  |
| ZA STL 121 | Enalapril | 5mg | DVA | India | Jun-23 | SIAB | Strips | Pass | Pass |  | Pass |  |
| NS PHO 222 | Enalapril | 5mg | DVA | India | Apr-22 | SIAB | Strips | Pass | Pass |  | Fail |  |
| MG MED 099 | Enalapril | 10mg | DVA | India | Jun-23 | Ordinary | Strips | Pass | Pass |  | Pass |  |
| ZA THO 13 | Enalapril | 10mg | DVA | India | Dec-21 | SIAB | Strips | Pass | Pass |  | Pass |  |
| ZA GHE 51 | Enalapril | 10mg | DVA | Tanzania | Mar-22 | Ordinary | Strips | Pass | Pass |  | Pass |  |
| NS DHO 197 | Enalapril | 10mg | DVA | India | Dec-21 | Ordinary | Strips | Pass | Pass |  | Pass |  |
| ZA MAT 165 | Glibenclamide | 5mg | DVA | Malawi | Feb-23 | Ordinary | Bottle | Pass | Pass |  | Pass |  |
| ZA THO 14 | Glibenclamide | 5mg | DVA | Malawi | Oct-22 | SIAB | Bottle | Pass | Pass |  | Pass |  |
| MG DHO 145 | Glibenclamide | 5mg | DVA | Malawi | Oct-22 | Ordinary | Bottle | Pass | Pass |  | Pass |  |
| MG NAY 54 | Glibenclamide | 5mg | DVA | India | Mar-22 | Ordinary | Bottle | Pass | Pass |  | Pass |  |
| MG NAM 62 | Glibenclamide | 5mg | DVA | Malawi | Oct-22 | Ordinary |  | Pass | Pass |  | Pass |  |
| MG MPO 110 | Glibenclamide | 5mg | DVA | India | Jun-22 | Ordinary | Strips | Pass | Pass |  | Pass |  |
| MG MGH 129 | Glibenclamide | 5mg | DVA | India | Mar-22 | SIAB | Strips | Pass | Pass |  | Pass |  |
| NS SAN 257 | Glibenclamide | 5mg | DVA | Malawi | Oct-22 | SIAB | Bottle | Pass | Pass |  | Pass |  |
| NS TRI 247 | Glibenclamide | 5mg | DVA | Malawi | Feb-23 | SIAB | Bottle | Pass | Pass |  | Pass |  |
| ZA DHO 26 | Glibenclamide | 5mg | DVA | Malawi | Oct-22 | Ordinary | Bottle | Pass | Pass |  | Pass |  |
| ZA STL 122 | Glibenclamide | 5mg | DVA | India | Jun-22 | SIAB | Strips | Pass | Pass |  | Pass |  |
| NS KAL 238 | Glibenclamide | 5mg | DVA | India | Jul-23 | SIAB | Strips | Pass | Pass |  | Pass |  |
| ZA CTL 037 | Glibenclamide | 5mg | DVA | Malawi | Oct-23 | Ordinary | Bottle | Pass | Pass |  | Pass |  |
| MG CHI 078 | Glibenclamide | 5mg | DVA | Malawi | Oct-22 | SIAB | Bottle | Pass | Pass |  | Pass |  |
| MG MED 101 | Glibenclamide | 5mg | DVA | Malawi | Feb-23 | Ordinary | Bottle | Pass | Pass |  | Pass |  |
| NS PHO 223 | Glibenclamide | 5mg | DVA | Malawi | Oct-22 | SIAB | Bottle | Pass | Pass |  | Pass |  |
| NS DHO 199 | Glibenclamide | 5mg | DVA | Malawi | Oct-22 | Ordinary | Bottle | Pass | Pass |  | Pass |  |
| ZA MAT 166 | Hydrochlorothiazide | 25mg | DVA | Kenya | Feb-24 | Ordinary | Bottle | Pass | Pass |  | Pass |  |
| MG GAW 136 | Hydrochlorothiazide | 250mg | DVA | India | Feb-24 | Ordinary | Bottle | Pass | Pass |  | Pass |  |
| MG NAM 87 | Hydrochlorothiazide | 25mg | DVA | Kenya | Oct-22 | Ordinary | Bottle | Pass | Pass |  | Pass |  |
| MG MPI 70 | Hydrochlorothiazide | 50mg | DVA | Kenya | Oct-21 | Ordinary | Bottle | Pass | Pass |  | Pass |  |
| MG MPO 111 | Hydrochlorothiazide | 25mg | DVA | Kenya | Feb-24 | Ordinary | Bottle | Pass | Pass |  | Pass |  |
| NS CHI 214 | Hydrochlorothiazide | 25mg | DVA | China | Nov-22 | Ordinary | Bottle | Pass | Pass |  | Pass |  |
| NS TRI 248 | Hydrochlorothiazide | 25mg | DVA | India | Feb-24 | SIAB | Bottle | Pass | Pass |  | Pass |  |
| ZA NKA 05 | Hydrochlorothiazide | 25mg | DVA | Netherlands | NOT INDICATED | Ordinary | Bottle | Pass | Pass |  | Pass |  |
| NS MBE 206 | Hydrochlorothiazide | 25mg | DVA | India | Jan-22 | SIAB | Bottle | Pass | Pass |  | Pass |  |
| NS TEN 231 | Hydrochlorothiazide | 25mg | DVA | India | Jan-22 | SIAB | Bottle | Pass | Pass |  | Pass |  |
| ZA STL 127 | Hydrochlorothiazide | 25mg | DVA | India | Feb-24 | SIAB | Bottle | Pass | Pass |  | Pass |  |
| NS PHO 225 | Hydrochlorothiazide | 25mg | DVA | India | Jan-22 | SIAB | Bottle | Pass | Pass |  | Pass |  |
| MG MED 102 | Hydrochlorothiazide | 25mg | DVA | India | Feb-24 | Ordinary | Bottle | Pass | Pass |  | Pass |  |
| NS DHO 198 | Hydrochlorothiazide | 25mg | DVA | India | Jan-22 | Ordinary | Bottle | Pass | Pass |  | Pass |  |
| MG CHI 084 | Metform | 850mg | DVA | India |  | SIAB | Strips | Pass | Pass | Pass | Pass | Pass |
| ZA MAT 167 | Metform | 500mg | DVA | Malawi | Dec-23 | Ordinary | Bottle | Pass | Pass | Pass | Pass | Pass |
| MG DHO 146 | Metform | 500mg | DVA | Malawi | Jul-22 | SIAB | Bottle | Pass | Pass | Pass | Pass | Pass |
| NS SAN 258 | Metform | 500mg | DVA | Malawi | Dec-21 | Ordinary | Bottle | Pass | Pass | Pass | Pass | Pass |
| NS TRI 249 | Metform | 500mg | DVA | India | May-23 | SIAB | Strips | Pass | Pass | Pass | Pass | Pass |
| NS PHO 224 | Metform | 500mg | DVA | Malawi | Dec-21 | SIAB | Bottle | Fail | Pass | Pass | Fail | Fail |
| NS KAL 239 | Metform | 500mg | DVA | India | Aug-22 | SIAB | Strips | Pass | Pass | Pass | Pass | Pass |
| ZA CTL 038 | Metform | 850mg | DVA | India | Dec-21 | Ordinary | Strips | Pass | Pass | Pass | Fail | Fail |
| MG CHI 079 | Metform | 500mg | DVA | Malawi | Mar-22 | SIAB | Bottle | Pass | Pass | Pass | Pass | Pass |
| MG MED 103 | Metform | 500mg | DVA | India | Oct-23 | Ordinary | Strips | Pass | Pass | Pass | Pass | Pass |
| NS DHO 200 | Metform | 850mg | DVA | India | Feb-23 | Ordinary | Strips | Pass | Pass | Pass | Pass | Pass |
| ZA MAK 191 | Metform | 500mg | DVA | Malawi | Dec-21 | SIAB | Bottle | Pass | Pass | Pass | Pass | Pass |
| ZA GHE 48 | Metform | 500mg | DVA | Malawi | Nov-23 | Ordinary | Bottle | Pass | Pass |  | Pass |  |
| ZA THO 15 | Metform | 500mg | DVA | India | Oct-24 | SIAB | Strips | Pass | Pass |  | Pass |  |
| ZA THO 16 | Metform | 850mg | DVA | India | Feb-23 | SIAB | Strips | Pass | Pass |  | Pass |  |
| ZA GHE 272 | Metform | 500mg | DVA | Malawi | May-24 | Ordinary | Bottle | Pass | Pass |  | Pass |  |
| ZA DHO 27 | Metform | 500mg | DVA | India | Dec-23 | Ordinary | Strips | Pass | Pass |  | Pass |  |
| ZA GHE 49 | Metform | 500mg | DVA | Kenya | Mar-24 | Ordinary | Bottle | Pass | Pass |  | Pass |  |
| ZA MAT 168 | Methyldopa | 250mg | DEA | Kenya | Jan-22 | Ordinary | Strips | Pass | Pass |  |  |  |
| MG DHO 147 | Methyldopa | 250mg | DEA | Malawi | Feb-24 | Ordinary | Strips | Pass | Pass |  |  |  |
| MG GAW 137 | Methyldopa | 250mg | DEA | Kenya | Oct-22 | Ordinary | Strips | Pass | Pass |  |  |  |
| NS TRI 250 | Methyldopa | 250mg | DEA | Kenya | Jan-22 | SIAB | Strips | Pass | Pass |  |  |  |
| ZA MAY 155 | Methyldopa | 250mg | DEA | Malawi | Apr-22 | Ordinary | Bottle | Pass | Pass |  |  |  |
| ZA DHO 28 | Methyldopa | 250mg | DEA | Malawi | Aug-22 | Ordinary | Bottle | Pass | Pass |  |  |  |
| MG MED 104 | Methyldopa | 250mg | DEA | Kenya | Jan-22 | Ordinary | Strips | Pass | Pass |  |  |  |
| ZA NKA 06 | Methyldopa | 25mg | DEA | India | Oct-22 | Ordinary | Strips | Pass | Pass |  |  |  |
| ZA CTL 039 | Methyldopa | 250mg | DEA | Malawi | Apr-22 | Ordinary | Bottle | Pass | Pass |  |  |  |
| NS TRI 245 | Artesunate/Amodiaquine | 185mg | DVA | Morocco | Feb-23 | SIAB | Strips | Pass | Pass |  |  |  |
| ZA CHA 296 | Lumefantrine Artemether | 140mg | HVA | India | Oct-22 | SIAB | Strips | Pass | Pass |  | Pass |  |
| ZA CHA 297 | Lumefantrine Artemether | 140mg | HVA | Switzerland | Aug-22 | SIAB | Strips | Pass | Pass |  | Pass |  |
| MG GAW 299 | Lumefantrine Artemether | 140mg | HVA | Switzerland | Aug-22 | Ordinary | Strips | Pass | Pass |  | Pass |  |
| MG GAW 300 | Lumefantrine Artemether | 140mg | HVA | India | Nov-23 | Ordinary | Strips | Pass | Pass |  | Pass |  |
| ZA MAY 275 | Lumefantrine Artemether | 140mg | HVA | India | Apr-24 | Ordinary | Strips | Pass | Pass |  | Pass |  |
| ZA MAY 276 | Lumefantrine Artemether | 140mg | HVA | India | Oct-22 | Ordinary | Strips | Pass | Pass |  | Pass |  |
| ZA MAK 278 | Lumefantrine Artemether | 140mg | HVA | Switzerland | Aug-22 | SIAB | Strips | Pass | Pass |  | Pass |  |
| ZA MAK 279 | Lumefantrine Artemether | 140mg | HVA | India | Nov-23 | SIAB | Strips | Pass | Pass |  | Pass |  |
| ZA MAC 280 | Lumefantrine Artemether | 140mg | HVA | India | Nov-23 | SIAB | Strips | Pass | Pass |  | Pass |  |
| ZA MAC 281 | Lumefantrine Artemether | 140mg | HVA | Switzerland | Aug-22 | SIAB | Strips | Pass | Pass |  | Pass |  |
| ZA GHE 270 | Lumefantrine Artemether | 140mg | HVA | India | Mar-24 | Ordinary | Strips | Pass | Pass |  | Pass |  |
| NS KAL 287 | Lumefantrine Artemether | 140mg | HVA | India | Sep-23 | SIAB | Strips | Pass | Pass |  | Pass |  |
| NS SAN 291 | Lumefantrine Artemether | 140ng | HVA | India | Oct-23 | SIAB | Strips | Pass | Pass |  | Pass |  |
| NS SAN 292 | Lumefantrine Artemether | 140ng | HVA | India | Dec-23 | SIAB | Strips | Pass | Pass |  | Pass |  |
| ZA CHA 298 | Lumefantrine Artemether | 140mg | HVA | India | Nov-23 | SIAB | Strips | Pass | Pass |  | Pass |  |
| NS DHO 203 | Lumefantrine Artemether | 140mg | HVA | Switzerland | May-22 | Ordinary | Strips | Pass | Pass |  | Pass |  |
| MG MPI 75 | Lumefantrine Artemether | 140mg | HVA | India | Aug-21 | Ordinary | Strips | Pass | Pass |  | Pass |  |
| NS CHI 218 | Lumefantrine Artemether | 140mg | HVA | India | Sep-21 | Ordinary | Strips | Pass | Pass |  | Pass |  |
| ZA MAY 157 | Lumefantrine Artemether | 140mg | HVA | Switzerland | Aug-22 | Ordinary | Strips | Pass | Pass |  | Pass |  |
| ZA MAY 158 | Lumefantrine Artemether | 140mg | HVA | India | Oct-23 | Ordinary | Strips | Pass | Pass |  | Pass |  |
| ZA MAY 159 | Lumefantrine Artemether | 140mg | HVA | Uganda | Dec-23 | Ordinary | Strips | Pass | Pass |  | Pass |  |
| MG NAY 57 | Lumefantrine Artemether | 140mg | HVA | India | Jan-23 | SIAB | Strips | Pass | Pass |  | Pass |  |
| MG MPI 73 | Lumefantrine Artemether | 140mg | HVA | India | Jul-22 | Ordinary | Strips | Pass | Pass |  | Pass |  |
| NS SAN 260 | Lumefantrine Artemether | 140mg | HVA | India | Jan-23 | SIAB | Strips | Pass | Pass |  | Pass |  |
| NS DHO 204 | Lumefantrine Artemether | 140mg | HVA | Switzerland | May-22 | Ordinary | Strips | Pass | Pass |  | Pass |  |
| MG NAY 58 | Lumefantrine Artemether | 140mg | HVA | Switzerland | Jun-20 | SIAB | Strips | Pass | Pass |  | Pass |  |
| ZA MAC 184 | Lumefantrine Artemether | 140mg | HVA | Switzerland | May-22 | SIAB | Strips | Pass | Pass |  | Pass |  |
| ZA MAC 185 | Lumefantrine Artemether | 140mg | HVA | Switzerland | Jul-22 | SIAB | Strips | Pass | Pass |  | Pass |  |
| MG MPO 114 | Lumefantrine Artemether | 140mg | HVA | Switzerland | Jul-22 | Ordinary | Strips | Pass | Pass |  | Pass |  |
| MG MGH 132 | Lumefantrine Artemether | 140mg | HVA | Switzerland | Jul-23 | Ordinary | Strips | Pass | Pass |  | Pass |  |
| NS TRI 252 | Lumefantrine Artemether | 140mg | HVA | Switzerland | May-22 | SIAB | Strips | Pass | Pass |  | Pass |  |
| NS MBE 209 | Lumefantrine Artemether | 140mg | HVA | Switzerland | Mar-22 | SIAB | Strips | Pass | Pass |  | Pass |  |
| ZA STL 124 | Lumefantrine Artemether | 140mg | HVA | Switzerland | Jul-22 | SIAB | Strips | Pass | Pass |  | Pass |  |
| MG CHI 083 | Lumefantrine Artemether | 140mg | HVA | Switzerland | May-22 | SIAB | Strips | Pass | Pass |  | Pass |  |
| NS PHO 228 | Lumefantrine Artemether | 140mg | HVA | Turkey | Mar-22 | SIAB | Strips | Pass | Pass |  | Pass |  |
| MG NAM 66 | Lumefantrine Artemether | 140mg | HVA | India | Oct-22 | SIAB | Strips | Pass | Pass |  | Pass |  |
| ZA CTL 042 | Lumefantrine Artemether | 140mg | HVA | India | Oct-22 | Ordinary | Strips | Pass | Pass |  | Pass |  |
| ZA NKA 08 | Lumefantrine Artemether | 140mg | HVA | India | Oct-22 | Ordinary | Strips | Pass | Pass |  | Pass |  |
| MG MPI 72 | Lumefantrine Artemether | 140mg | HVA | Switzerland | Jul-22 | Ordinary | Strips | Pass | Pass |  | Pass |  |
| MG NAM 67 | Lumefantrine Artemether | 140mg | HVA | Switzerland | Mar-22 | SIAB | Strips | Pass | Pass |  | Pass |  |
| NS CHI 216 | Lumefantrine Artemether | 140mg | HVA | Switzerland | May-22 | Ordinary | Strips | Pass | Pass |  | Pass |  |
| NS KAL 241 | Lumefantrine Artemether | 140mg | HVA | Switzerland | May-22 | SIAB | Strips | Pass | Pass |  | Pass |  |
| MG MPI 74 | Lumefantrine Artemether | 140mg | HVA | India | Aug-22 | Ordinary | Strips | Pass | Pass |  | Pass |  |
| ZA MAC 187 | Lumefantrine Artemether | 140mg | HVA | India | Aug-22 | SIAB | Strips | Pass | Pass |  | Pass |  |
| NS CHI 217 | Lumefantrine Artemether | 140mg | HVA | India | Jul-22 | Ordinary | Strips | Pass | Pass |  | Pass |  |
| ZA STL 125 | Lumefantrine Artemether | 140mg | HVA | India | Jun-23 | SIAB | Strips | Pass | Pass |  | Pass |  |
| ZA MAT 172 | Lumefantrine Artemether | 140mg | HVA | India | Jun-23 | Ordinary | Strips | Pass | Pass |  | Pass |  |
| MG NAY 56 | Lumefantrine Artemether | 140mg | HVA | Uganda | Dec-23 | SIAB | Strips | Pass | Pass |  | Pass |  |
| ZA CHA 178 | Lumefantrine Artemether | 140mg | HVA | Uganda | Jun-23 | SIAB | Strips | Pass | Pass |  | Pass |  |
| NS DHO 211 | Lumefantrine Artemether | 140mg | HVA | Uganda | Jun-23 | Ordinary | Strips | Pass | Pass |  | Pass |  |
| NS PHO 229 | Lumefantrine Artemether | 140mg | HVA | Uganda | Jun-23 | SIAB | Strips | Pass | Pass |  | Pass |  |
| NS PHO 230 | Lumefantrine Artemether | 140mg | HVA | Uganda | Feb-23 | SIAB | Strips | Pass | Pass |  | Pass |  |
| ZA THO 18 | Lumefantrine Artemether | 240mg | HVA | India | Mar-23 | SIAB | Strips | Pass | Pass |  | Pass |  |
| MG DHO 150 | Lumefantrine Artemether | 140mg | HVA | India | Oct-23 | Ordinary | Strips | Pass | Pass |  | Pass |  |
| ZA CHA 180 | Lumefantrine Artemether | 140mg | HVA | India | Sep-23 | SIAB | Strips | Pass | Pass |  | Pass |  |
| MG MPO 113 | Lumefantrine Artemether | 140mg | HVA | India | Apr-23 | Ordinary | Strips | Pass | Pass |  | Pass |  |
| MG MGH 131 | Lumefantrine Artemether | 140mg | HVA | India | Sep-23 | Ordinary | Strips | Pass | Pass |  | Pass |  |
| NS SAN 261 | Lumefantrine Artemether | 140mg | HVA | India | Sep-23 | SIAB | Strips | Pass | Pass |  | Pass |  |
| NS SAN 262 | Lumefantrine Artemether | 140mg | HVA | Uganda | May-23 | SIAB | Strips | Pass | Pass |  | Pass |  |
| NS MBE 208 | Lumefantrine Artemether | 140mg | HVA | India | Apr-23 | SIAB | Strips | Pass | Pass |  | Pass |  |
| ZA DHO 30 | Lumefantrine Artemether | 140mg | HVA | China | Oct-23 | Ordinary | Strips | Pass | Pass |  | Pass |  |
| NS KAL 243 | Lumefantrine Artemether | 140mg | HVA | India | Sep-23 | SIAB | Strips | Pass | Pass |  | Pass |  |
| MG CHI 081 | Lumefantrine Artemether | 140mg | HVA | India | Apr-23 | SIAB | Strips | Pass | Pass |  | Pass |  |
| ZA MAT 173 | Lumefantrine Artemether | 140mg | HVA | Uganda | Nov-23 | Ordinary | Strips | Pass | Pass |  | Pass |  |
| MG MPO 115 | Lumefantrine Artemether | 140mg | HVA | Uganda | Nov-23 | Ordinary | Strips | Pass | Pass |  | Pass |  |
| ZA THO 20 | Lumefantrine Artemether | 240mg | HVA | Uganda | Dec-23 | SIAB | Strips | Pass | Pass |  | Pass |  |
| ZA MAC 186 | Lumefantrine Artemether | 140mg | HVA | Uganda | Jun-23 | SIAB | Strips | Pass | Pass |  | Pass |  |
| NS TRI 253 | Lumefantrine Artemether | 140mg | HVA | Uganda | Dec-23 | SIAB | Strips | Pass | Pass |  | Pass |  |
| NS KAL 242 | Lumefantrine Artemether | 140mg | HVA | Uganda | May-23 | SIAB | Strips | Pass | Pass |  | Pass |  |
| NS PHO 227 | Lumefantrine Artemether | 140mg | HVA | India | Mar-23 | SIAB | Strips | Pass | Pass |  | Pass |  |
| MG NAM 90 | Lumefantrine Artemether | 140mg | HVA | India | Dec-23 | Ordinary | Strips | Pass | Pass |  | Pass |  |
| NS MBE 210 | Lumefantrine Artemether | 140mg | HVA | Uganda | Jun-23 | SIAB | Strips | Pass | Pass |  | Pass |  |
| NS TEN 235 | Lumefantrine Artemether | 140mg | HVA | Uganda | Dec-23 | SIAB | Strips | Pass | Pass |  | Pass |  |
| MG MED 106 | Lumefantrine Artemether | 560mg | HVA | India | Apr-23 | Ordinary | Strips | Pass | Pass |  | Pass |  |
| ZA CTL 043 | Lumefantrine Artemether | 140mg | HVA | Uganda | Nov-23 | Ordinary | Strips | Pass | Pass |  | Pass |  |
| NS DHO 212 | Lumefantrine Artemether | 140mg | HVA | Uganda | May-23 | Ordinary | Strips | Pass | Pass |  | Pass |  |
| MG DHO 149 | Lumefantrine Artemether | 140mg | HVA | India | Sep-23 | Ordinary | Strips | Pass | Pass |  | Pass |  |
| MG NAM 65 | Lumefantrine Artemether | 140mg | HVA | India | Nov-23 | SIAB | Strips | Pass | Pass |  | Pass |  |
| ZA CTL 041 | Lumefantrine Artemether | 140mg | HVA | India | Nov-23 | Ordinary | Strips | Pass | Pass |  | Pass |  |
| MG CHI 082 | Lumefantrine Artemether | 140mg | HVA | India | Nov-23 | SIAB | Strips | Pass | Pass |  | Pass |  |
| MG NAM 89 | Lumefantrine Artemether | 140mg | HVA | India | Nov-23 | Ordinary | Strips | Pass | Pass |  | Pass |  |
| MG NAM 91 | Lumefantrine Artemether | 140mg | HVA | India | Nov-23 | Ordinary | Strips | Pass | Pass |  | Pass |  |
| MG NAM 64 | Lumefantrine Artemether | 140mg | HVA | India | Sep-23 | SIAB | Strips | Pass | Pass |  | Pass |  |
| MG MGH 133 | Lumefantrine Artemether | 140mg | HVA | India | Nov-23 | Ordinary | Strips | Pass | Pass |  | Pass |  |
| NS TRI 254 | Lumefantrine Artemether | 140mg | HVA | India | Nov-23 | SIAB | Strips | Pass | Pass |  | Pass |  |
| NS TEN 233 | Lumefantrine Artemether | 140mg | HVA | India | Oct-23 | SIAB | Strips | Pass | Pass |  | Pass |  |
| NS TEN 234 | Lumefantrine Artemether | 140mg | HVA | India | Sep-23 | SIAB | Strips | Pass | Pass |  | Pass |  |
| ZA STL 126 | Lumefantrine Artemether | 140mg | HVA | India | Sep-23 | SIAB | Strips | Pass | Pass |  | Pass |  |
| ZA NKA 07 | Lumefantrine Artemether | 140mg | HVA | Switzerland |  | Ordinary | Strips | Pass | Pass |  | Pass |  |
| ZA THO 19 | Lumefantrine Artemether | 240mg | HVA | Switzerland | Aug-22 | SIAB | Strips | Pass | Pass |  | Pass |  |
| MG GAW 141 | Lumefantrine Artemether | 140mg | HVA | Switzerland | Aug-22 | Ordinary | Strips | Pass | Pass |  | Pass |  |
| MG MGH 134 | Lumefantrine Artemether | 140mg | HVA | Switzerland | May-22 | SIAB | Strips | Pass | Pass |  | Pass |  |
| MG NAM 92 | Lumefantrine Artemether | 140mg | HVA | India | Oct-22 | Ordinary | Strips | Pass | Pass |  | Pass |  |
| MG GAW 139 | Lumefantrine Artemether | 140mg | HVA | India | Oct-22 | Ordinary | Strips | Pass | Pass |  | Pass |  |
| MG DHO 151 | Lumefantrine Artemether | 140mg | HVA | Switzerland | May-22 | Ordinary | Strips | Pass | Pass |  | Pass |  |
| ZA CHA 179 | Lumefantrine Artemether | 140mg | HVA | Switzerland | Aug-22 | SIAB | Strips | Pass | Pass |  | Pass |  |
| NS TEN 232 | Lumefantrine Artemether | 140mg | HVA | Switzerland | May-22 | SIAB | Strips | Pass | Pass |  | Pass |  |
| MG GAW 140 | Lumefantrine Artemether | 140mg | HVA | India | Oct-23 | Ordinary | Strips | Pass | Pass |  | Pass |  |
| ZA THO 21 | Lumefantrine Artemether | 240mg | HVA | India | Dec-20 | SIAB | Strips | Pass | Pass |  | Pass |  |
| MG MED 105 | Quinine sulfate | 300mg | DVA | India | Jul-22 | SIAB | Bottle | Pass | Pass |  | Pass |  |
| ZA MAY 273 | Sulfadoxine pyrimethamine | 525mg | HVA | China | May-23 | Ordinary | Bottle | Pass | Fail |  | Pass | Fail |
| ZA MAY 274 | Sulfadoxine pyrimethamine | 525mg | HVA | China | Mar-24 | Ordinary | Bottle | Pass | Pass |  | Pass |  |
| ZA GHE 271 | Sulfadoxine pyrimethamine | 525mg | HVA | Tanzania | Jun-25 | Ordinary | Bottle | Pass | Pass |  | Pass |  |
| ZA MAT 169 | Sulfadoxine pyrimethamine | 325mg | HVA | China | May-22 | Ordinary | Bottle | Pass | Pass |  | Pass | Fail |
| ZA THO 17 | Sulfadoxine pyrimethamine | 525mg | HVA | China | May-22 | SIAB | Bottle | Pass | Pass |  | Pass | Fail |
| MG DHO 148 | Sulfadoxine pyrimethamine | 525mg | HVA | China | Dec-22 | Ordinary | Bottle | Pass | Pass |  | Pass | Fail |
| MG GAW 138 | Sulfadoxine pyrimethamine | 525mg | HVA | China | Oct-23 | Ordinary | Bottle | Pass | Pass |  | Pass | Fail |
| ZA GHE 50 | Sulfadoxine pyrimethamine | 525mg | HVA | Tanzania | Aug-22 | Ordinary | Bottle | Pass | Pass |  | Pass | Fail |
| ZA CHA 177 | Sulfadoxine pyrimethamine | 525mg | HVA | China | Dec-21 | SIAB | Bottle | Pass | Pass |  | Pass | Fail |
| MG NAM 88 | Sulfadoxine pyrimethamine | 525mg | HVA | China | May-23 | Ordinary | Bottle | Pass | Pass |  | Pass |  |
| MG NAY 55 | Sulfadoxine pyrimethamine | 525mg | HVA | India | Dec-22 | SIAB | Bottle | Pass | Pass |  | Pass |  |
| MG MPI 71 | Sulfadoxine pyrimethamine | 525mg | HVA | China | Dec-22 | Ordinary | Bottle | Pass | Pass |  | Pass |  |
| MG NAM 63 | Sulfadoxine pyrimethamine | 525mg | HVA | China | Dec-22 | SIAB | Bottle | Pass | Pass |  | Pass |  |
| ZA MAC 183 | Sulfadoxine pyrimethamine | 525mg | HVA | China | Dec-22 | SIAB | Bottle | Pass | Pass |  | Pass |  |
| MG MPO 112 | Sulfadoxine pyrimethamine | 525mg | HVA | China | Dec-21 | Ordinary | Bottle | Pass | Pass |  | Pass |  |
| MG MGH 130 | Sulfadoxine pyrimethamine | 525mg | HVA | China | Dec-21 | Ordinary | Bottle | Pass | Pass |  | Pass | Fail |
| NS SAN 259 | Sulfadoxine pyrimethamine | 525mg | HVA | China | Dec-22 | SIAB | Bottle | Pass | Pass |  | Pass |  |
| NS CHI 215 | Sulfadoxine pyrimethamine | 525mg | HVA | China | May-22 | Ordinary | Bottle | Pass | Fail |  | Pas | Fail |
| NS TRI 251 | Sulfadoxine pyrimethamine | 525mg | HVA | China | Dec-22 | SIAB | Bottle | Pass | Pass |  | Pass |  |
| NS MBE 207 | Sulfadoxine pyrimethamine | 525mg | HVA | China | Dec-22 | SIAB | Bottle | Pass | Pass |  | Pass |  |
| ZA DHO 29 | Sulfadoxine pyrimethamine | 525mg | HVA | China | Dec-22 | Ordinary | Bottle | Pass | Pass |  | Pass |  |
| ZA STL 123 | Sulfadoxine pyrimethamine | 525mg | HVA | China | May-23 | SIAB | Bottle | Pass | Pass |  | Pass |  |
| NS PHO 226 | Sulfadoxine pyrimethamine | 525mg | HVA | China | Dec-22 | SIAB | Bottle | Pass | Pass |  | Pass |  |
| NS KAL 240 | Sulfadoxine pyrimethamine | 525mg | HVA | China | May-22 | SIAB | Bottle | Pass | Pass |  | Pass |  |
| ZA CTL 040 | Sulfadoxine pyrimethamine | 525mg | HVA | China | Dec-22 | Ordinary | Bottle | Pass | Pass |  | Pass |  |
| MG CHI 080 | Sulfadoxine pyrimethamine | 525mg | HVA | China | Dec-22 | SIAB | Bottle | Pass | Pass |  | Pass |  |
| ZA MAY 156 | Sulfadoxine pyrimethamine | 525mg | HVA | China | Apr-22 | Ordinary | Bottle | Pass | Pass |  | Pass |  |
| ZA CHA 295 | Sulfadoxine pyrimethamine | 525mg | HVA | China | Dec-22 | SIAB | Bottle | Pass | Fail |  | Pass | Fail |

a The Malawi Essential Medicines List (MEML) of 2015 specifies the level of health institution at which the medicine is normally permitted for use: H = at health centre, district hospital and central hospital levels; D = at district hospital and central hospital levels only; C = at central hospital level only. N = level of use not specified. The ‘therapeutical priority’ code categorizes medicines based on therapeutic importance of each medicine by the use of: V = vital medicines which are potentially life-saving, of major public health relevance and having significant withdraw side-effects, E = essential medicines which are effective against less severe, but nonetheless significant forms of illness; N = non-essential medicines which are used for minor self-limiting illness and are often of questionable efficacy. The third categorization of ‘procurement system’ has two codes: ‘A’= medicines required by a large number of patients as such to be routinely procured and stocked by CMST; and ‘B’= medicines required for a limited number of patients and not routinely stocked by CMST).
