# Supplementary material for: Burden of and factors associated with poor quality antibiotic, antimalarial, antihypertensive and antidiabetic medicines in Malawi
Source: PLoS One. 2022 Dec 27;17(12):e0279637. doi: 10.1371/journal.pone.0279637 (PMC9794066; doi:10.1371/journal.pone.0279637)
Supplement: S2 Table — (DOCX) [file pone.0279637.s002.docx]

**S2 Table. List and description of results of medicines that failed various laboratory tests and reasons for non-compliance**

| **Medicine (INN)** | **Sample ID** | **Type of Sampling Site** |  | **Stated Country of origin** | **Primary packaging** | **Visual Inspection** | **TLC** | **Disintegration** | **Dissolution** | **Assay**  **(HPLC/UV-Vis)** | **Reason for non-compliance (reference)** |
| --- | --- | --- | --- | --- | --- | --- | --- | --- | --- | --- | --- |
| **SF medicines with assay deviations not less than 80%( non- extreme deviations)** | | | | | | | | | | | |
| Atenolol | NS-DHO 201 | Public |  | Malawi | Bottle | Tablet discoloration | Compliant | Compliant | 56.9% | 89.9%  (UV/Vis) | Low API content and dissolution (USP-38 NF 33) |
| Atenolol | ZA CHA 175 | Public |  | Malawi | Bottle | Tablet discoloration | Compliant | Compliant | 80.0% | 83.9%  (UV/Vis) | Low API content (USP-38 NF 33) |
| Atenolol | ZA CTL 034 | Public |  | Malawi | Bottle | Tablet discoloration | Compliant | Compliant | 51.2% | 94.8%  (UV/Vis) | Low dissolution (USP-38 NF 33) |
| Atenolol | ZA STL 119 | Faith-based |  | Malawi | Bottle | Tablet discoloration | Compliant | Compliant | 53.0% | 90.6%  (UV/Vis) | Low dissolution (USP-38 NF 33) |
| Atenolol | ZA DHO 23 | Public |  | Malawi | Bottle | Tablet discoloration | Compliant | Compliant | 26.8% | 87.7%  (UV/Vis) | Low API content and dissolution (USP-38 NF 33) |
| Atenolol | ZA THO 10 | Public |  | Malawi | Bottle | Tablet discoloration | Compliant | Compliant | 31.4% | 89.9%  (UV/Vis) | Low API content and dissolution (USP-38 NF 33) |
| Atenolol | ZA MAT 161 | Public |  | Malawi | Bottle | Compliant | Compliant | Compliant | 72.5% | 81.6%  (UV/Vis) | Low API content (USP-38 NF 33) |
| Atenolol | MG DHO 143 | Public |  | India | Strips | Compliant | Compliant | Compliant | 77.8% | 88.8%  (UV/Vis) | Low API content (USP-38 NF 33) |
| Metformin | NS PHO 224 | Public |  | Malawi | Bottle | Tablet discoloration | Compliant | Compliant | 77.1% | 87.7%  (UV/Vis) | Low API content (USP-38 NF 33) |
| Metformin | ZA CTL 038 | Public |  | India | Strips | Compliant | Compliant | Compliant | 71.7% | 84.1%  (UV/Vis) | Low API content (USP-38 NF 33) |
| Amoxicillin | ZA THO 09 | Public |  | Malawi | Bottle | Compliant | Compliant | Compliant | 65.0% | 87.9%  (HPLC) | Low API content (USP-38 NF 33) |
| Amoxicillin | MG NAY 52 | Public |  | Malawi | Bottle | Compliant | Compliant | Non-compliant | 59.3% | 86.6%  (UV/Vis) | Low API content and dissolution (USP-38 NF 33) |
| Amoxicillin | MG MPI 68 | Public |  | India | Bottle | Compliant | Compliant | Non-compliant | 57.3% | 84.9%  (HPLC) | Low API content and dissolution (USP-38 NF 33) |
| Amoxicillin | ZA STI 118 | Faith-based |  | Kenya | Bottle | Compliant | Compliant | Non-compliant | 52.5% | 82.9%  (HPLC) | Low API content and dissolution (USP-38 NF 33) |
| Amoxicillin | NS SAN 244 | Public |  | Kenya | Bottle | Compliant | Compliant | Non-compliant | 66.3% | 84.7%  (HPLC) | Low API content and dissolution (USP-38 NF 33) |
| Ciprofloxacin | ZA CHA 294 | Public |  | Malawi | Bottle | Compliant | Non-compliant | Non-compliant | 50.6% | 83.5%  (HPLC) | Low API content and dissolution (USP-38 NF 33) |
| Ciprofloxacin | NS MBE 288 | Public |  | Malawi | Bottle | Compliant | Non-compliant | Non-compliant | 64.8% | 86.0%  (HPLC) | Low API content and dissolution (USP-38 NF 33) |
| Ciprofloxacin | NS PHO 290 | Public |  | Malawi | Bottle | Compliant | Non-compliant | Non-compliant | 56.6% | 74.3%  (HPLC) | Low API content and dissolution (USP-38 NF 33) |
| Ciprofloxacin | NS MBE 289 | Public |  | India | Strips | Compliant | Compliant | Compliant | 72.3% | 88.2%  (HPLC) | Low API content (USP-38 NF 33) |
| Ciprofloxacin | NS TRI 286 | Faith-based |  | India | Strips | Compliant | Compliant | Compliant | 73.7% | 81.8%  (HPLC) | Low API content (USP-38 NF 33) |
| Ciprofloxacin | NS MBE 301 | Public |  | Malawi | Bottle | Compliant | Compliant | Compliant | 70.1% | 77.5%  (HPLC) | Low API content (USP-38 NF 33) |
| Ciprofloxacin | NS DHO 257 | Public |  | Malawi | Bottle | Compliant | Non-compliant | Non-compliant | 57.6% | 91.5%  (HPLC) | Low dissolution (USP-38 NF 33) |
| **SF medicines assay deviations below 80% or above 120% ( extreme deviations)** | | | | | | | | | | | |
| SP (pyrimethamine (1) and Sulfadoxine (2) | MG MGH 130 (1) | Public |  | China | Bottle | Compliant | Non-compliant | Compliant | NA | 191.2%  (HPLC) | High API content (USP-38 NF 33) |
|  | MG MGH 130 (2) | Public |  | China | Bottle | Compliant | Compliant | Compliant | NA | 58.1%  (HPLC) | Low API content (USP-38 NF 33) |
| SP (pyrimethamine (1) and Sulfadoxine (2) | ZA MAK 169 (1) | Public |  | China | Bottle | Compliant | Compliant | Compliant | NA | 175.3  (HPLC) | High API content (USP-38 NF 33) |
|  | ZA MAK 169 (2) | Public |  | China | Bottle | Compliant | Compliant | Compliant | NA | 57.1%  (HPLC) | Low API content (USP-38 NF 33) |
| SP (pyrimethamine (1) and Sulfadoxine (2) | ZA MAY 273 (1) | Faith-based |  | China | Bottle | Compliant | Compliant | Compliant | NA | 197.2%  (HPLC) | High API content (USP-38 NF 33) |
|  | ZA MAY 273 (2) | Faith-based |  | China | Bottle | Compliant | Compliant | Compliant | NA | 57.3%  (HPLC) | Low API content (USP-38 NF 33) |
| SP (pyrimethamine (1) and Sulfadoxine (2) | ZA CHI 215 (1) | Public |  | China | Bottle | Compliant | Compliant | Non-compliant | NA | 166.9%  (HPLC) | High API content (USP-38 NF 33) |
|  | ZA CHI 215 (2) | Public |  | China | Bottle | Compliant | Compliant | Non-compliant | NA | 63.0%  (HPLC) | Low API content (USP-38 NF 33) |
| SP (pyrimethamine (1) and Sulfadoxine (2) | ZA CHA 295 (1) | Public |  | China | Bottle | Compliant | Compliant | Non-compliant | NA | 168.5%  (HPLC) | High API content (USP-38 NF 33) |
|  | ZA CHA 295 (2) | Public |  | China | Bottle | Compliant | Compliant | Non-compliant | NA | 55.3%  (HPLC) | Low API content (USP-38 NF 33) |
| SP (pyrimethamine (1) and Sulfadoxine (2) | ZA THO 17 (1) | Public |  | China | Bottle | Compliant | Compliant | Compliant | NA | 165.3%  (HPLC) | High API content (USP-38 NF 33) |
|  | ZA THO 17 (2) | Public |  | China | Bottle | Compliant | Compliant | Compliant | NA | 77.2%  (HPLC) | Low API content (USP-38 NF 33) |
| SP (pyrimethamine (1) and Sulfadoxine (2) | MG DHO 148 (1) | Public |  | China | Bottle | Compliant | Compliant | Compliant | NA | 166.9%  (HPLC) | High API content (USP-38 NF 33) |
|  | MG DHO 148 (2) | Public |  | China | Bottle | Compliant | Compliant | Compliant | NA | 67.5%  (HPLC) | Low API content (USP-38 NF 33) |
| SP (pyrimethamine (1) and Sulfadoxine (2) | MG GAW 138 (1) | Faith-based |  | China | Bottle | Compliant | Compliant | Compliant | NA | 150.2%  (HPLC) | High API content (USP-38 NF 33) |
|  | MG GAW 138 (2) | Faith-based |  | China | Bottle | Compliant | Compliant | Compliant | NA | 77.1%  (HPLC) | Low API content (USP-38 NF 33) |
| SP (pyrimethamine (1) and Sulfadoxine (2) | MG NAM 88 (1) | Faith-based |  | China | Bottle | Compliant | Compliant | Compliant | NA | 171.8%  (HPLC) | High API content (USP-38 NF 33) |
|  | MG NAM 89 (2) | Faith-based |  | China | Bottle | Compliant | Compliant | Compliant | NA | 66.3% | Low API content (USP-38 NF 33) |
| SP (pyrimethamine (1) and Sulfadoxine (2) | ZA CHA 177 (1) | Public |  | China | Bottle | Compliant | Compliant | Compliant | NA | 153.3% | High API content (USP-38 NF 33) |
|  | ZA CHA 177 (2) | Public |  | China | Bottle | Compliant | Compliant | Compliant | NA | 67.6% | Low API content (USP-38 NF 33) |
| **SF not confirmed by pharmacopeial analysis** | | | | | | | | | | | |
| Flucloxacillin | ZA MAT 164 | Public |  | Kenya | Strips | Compliant | NA | Non-compliant | NA | NA | Capsule disintergration time >30 min (Mini lab) |
| Flucloxacillin | ZA GHE 47 | Private |  | Kenya | Strips | Compliant | NA | Non-compliant | NA | NA | Capsule disintegration time >30 min (Mini lab) |
|  |  |  |  |  |  |  |  |  |  |  |  |
| Flucloxacillin | ZA MAC 182 | Public |  | India | Strips | Compliant | NA | Non-compliant | NA | NA | Capsule disintegration time >30 min (Mini lab) |
| Flucloxacillin | MG MED 100 | Private |  | England | Strips | Compliant | NA | Non-compliant | NA | NA | Capsule disintegration time >30 min (Mini lab) |
| Amlodipine | NS KAL 236 | Faith-based |  | India | Strips | Compliant | Non-compliant | Compliant | NA | NA | Low API content observed by spot size (Mini lab) |
| Enalapril | NS PHO 222 | Public |  | India | Strips | Compliant | Non-compliant | Compliant | NA | NA | Low API content observed by spot size (Mini lab) |
